# Evaluation of pathogen detection from clinical samples by real-time polymerase chain reaction using a sepsis pathogen DNA detection kit

**DOI:** 10.1186/cc9234

**Published:** 2010-08-24

**Authors:** Katsunori Yanagihara, Yuko Kitagawa, Masao Tomonaga, Kunihiro Tsukasaki, Shigeru Kohno, Masafumi Seki, Hisashi Sugimoto, Takeshi Shimazu, Osamu Tasaki, Asako Matsushima, Yasuo Ikeda, Shinichiro Okamoto, Naoki Aikawa, Shingo Hori, Hideaki Obara, Akitoshi Ishizaka, Naoki Hasegawa, Junzo Takeda, Shimeru Kamihira, Kazuyuki Sugahara, Seishi Asari, Mitsuru Murata, Yoshio Kobayashi, Hiroyuki Ginba, Yoshinobu Sumiyama, Masaki Kitajima

**Affiliations:** 1 Department of Laboratory Medicine, Nagasaki University School of Medicine, 1-7-1 Sakamoto, Nagasaki City, Nagasaki 852-8501, Japan; 2 Department of Surgery, Keio University School of Medicine, 35, Shinanomachi, Shinjuku-ku, Tokyo, 160-8582, Japan; 3 Department of Hematology, Nagasaki University School of Medicine, 1-7-1 Sakamoto, Nagasaki City, Nagasaki 852-8501, Japan; 4 Second Department of Internal Medicine, Nagasaki University School of Medicine, 1-7-1 Sakamoto, Nagasaki City, Nagasaki 852-8501, Japan; 5 Department of Traumatology and Acute Critical Medicine, Osaka University Graduate School of Medicine, 2-15, Yamadaoka, Suita city, Osaka, 565-0871, Japan; 6 Department of Medicine, Keio University School of Medicine, 35, Shinanomachi, Shinjuku-ku, Tokyo, 160-8582, Japan; 7 Department of Emergency & Critical Care Medicine, Keio University School of Medicine, 35, Shinanomachi, Shinjuku-ku, Tokyo, 160-8582, Japan; 8 Department of Anesthesiology, Keio University School of Medicine, 35, Shinanomachi, Shinjuku-ku, Tokyo, 160-8582, Japan; 9 Department of Laboratory Medicine, Osaka University Graduate School of Medicine, 2-15, Yamadaoka, Suita city, Osaka, 565-0871, Japan; 10 Department of Laboratory Medicine, Keio University School of Medicine, 35, Shinanomachi, Shinjuku-ku, Tokyo, 160-8582, Japan; 11 Roche Diagnostics K.K. Shiba 2-Chome Minato-Ku, Tokyo, 105-0014, Japan; 12 Third Department of Surgery, Toho University School of Medicine, Ohashi Medical Center, 2-17-6 Ohashi, Meguro-ku, Tokyo 153-8515, Japan

## Abstract

**Introduction:**

Sepsis is a serious medical condition that requires rapidly administered, appropriate antibiotic treatment. Conventional methods take three or more days for final pathogen identification and antimicrobial susceptibility testing. We organized a prospective observational multicenter study in three study sites to evaluate the diagnostic accuracy and potential clinical utility of the Septi*Fast *system, a multiplex pathogen detection system used in the clinical setting to support early diagnosis of bloodstream infections.

**Methods:**

A total of 212 patients, suspected of having systemic inflammatory response syndrome (SIRS) caused by bacterial or fungal infection, were enrolled in the study. From these patients, 407 blood samples were taken and blood culture analysis was performed to identify pathogens. Whole blood was also collected for DNA Detection Kit analysis immediately after its collection for blood culture. The results of the DNA Detection Kit, blood culture and other culture tests were compared. The chosen antimicrobial treatment in patients whose samples tested positive in the DNA Detection Kit and/or blood culture analysis was examined to evaluate the effect of concomitant antibiotic exposure on the results of these analyses.

**Results:**

Septi*Fast *analysis gave a positive result for 55 samples, while 43 samples were positive in blood culture analysis. The DNA Detection Kit identified a pathogen in 11.3% (45/400) of the samples, compared to 8.0% (32/400) by blood culture analysis. Twenty-three pathogens were detected by Septi*Fast *only; conversely, this system missed five episodes of clinically significant bacteremia (Methicillin-resistant *Staphylococcus aureus *(MRSA), 2; *Pseudomonas aeruginosa*, 1; *Klebsiella spp*, 1; *Enterococcus faecium*, 1). The number of samples that tested positive was significantly increased by combining the result of the blood culture analysis with those of the DNA Detection Kit analysis (*P *= 0.01). Among antibiotic pre-treated patients (prevalence, 72%), Septi*Fast *analysis detected more bacteria/fungi, and was less influenced by antibiotic exposure, compared with blood culture analysis (*P *= 0.02).

**Conclusions:**

This rapid multiplex pathogen detection system complemented traditional culture-based methods and offered some added diagnostic value for the timely detection of causative pathogens, particularly in antibiotic pre-treated patients. Adequately designed intervention studies are needed to prove its clinical effectiveness in improving appropriate antibiotic selection and patient outcomes.

## Introduction

Sepsis is a serious medical condition frequently found in transplant patients, in patients with hematological neoplasms or in patients admitted to the intensive care unit (ICU) after surgery. Rapid pathogen identification and appropriate chemotherapy are important to improve patient prognoses. In the United States, more than 750,000 cases of sepsis are reported annually [1]. The fatality rate is 28% to 50% for severe sepsis and as high as 90% when the causative agent is *Aspergillus *[1-3]. For most cases of suspected sepsis, blood culture analysis is performed for pathogen detection, and empirical treatment with broad-spectrum antibiotics is immediately started without waiting for the result of pathogen identification. This is because, in many cases, positive pathogen identification, and pathogen drug sensitivity analysis, using blood culture analysis, requires from three days to a week for common bacteria and a few weeks for fungi [4,5]. Therefore, choosing the appropriate antibiotic chemotherapy according to evidence-based medicine (EBM) is currently difficult in many sepsis cases. Moreover, in some cases, inappropriate antibiotic selection not only annuls the effects of chemotherapy but also promotes the emergence of drug-resistant bacteria.

Because of these problems with sepsis diagnosis, highly sensitive sepsis-pathogen detection methods using nucleic acid amplification techniques such as PCR have been recently studied for the purpose of rapid testing and the subsequent choosing of appropriate chemotherapy. However, the development of a diagnostic reagent to simultaneously detect a wide range of sepsis pathogens has been difficult using conventional genetic technology.

A new assay, termed Septi*Fast *(Roche Diagnostics, Mannheim, Germany), enables rapid, multiplex testing for micro-organisms using a real-time polymerase chain reaction that is coupled to melting curve analysis. This kit can identify up to 25 organisms from four different microbial groups, in a single sample, in about 4.5 hours [6].

We organized a clinical performance research group to investigate the potential clinical utility of Septi*Fast *analysis by comparing with those obtained using the currently used routine blood culture analysis. We also compared the effect of antibiotic treatment on detection of pathogens by DNA Detection Kit and blood culture analysis, and we analyzed the number of pathogens that could be detected when the results of both assay methods were combined.

## Materials and methods

We conducted a prospective multicenter study in Japan of Septi*Fast *(Roche Diagnostics GmbH, Mannheim, Germany) analysis, which detects sepsis pathogens in whole blood. Septi*Fast *is currently used as an *in vitro *diagnostic reagent in Europe. Table 1 lists the bacteria and fungi that are detectable by DNA Detection Kit analysis. When *S. aureus *was detected, Septi*Fast *mecA kit was used to confirm whether this *S. aureus *was MRSA or not.

**Table 1:** Pathogens listed in the Septi*Fast *PCR menu

Gram-positive bacteria	Gram-negative bacteria	Fungi
*Staphylococcus aureus*	*Escherichia coli*	*Candida albicans*
*Coagulase negative Staphylococcus*	*Klebsiella (pneumoniae/oxyt.)*	*Candida tropicalis*
*Streptococcus pneumonia*	*Serratia marcescens*	*Candida parapsilosis*
*Streptococcus spp*.	*Enterobacter (cloacae/aerog.)*	*Candida krusei*
*Enterococcus faecium*	*Proteus mirabilis*	*Candida glabrata*
*Enterococcus faecalis*	*Pseudomonas aeruginosa*	*Aspergillus (fumigatus)*
	*Acinetobacter baumanii*	
	*Stenotrophomonas maltophilia*	

Drug-resistant bacteria. mecA (MRSA).

This study was conducted at Keio University, Osaka University and Nagasaki University from May 2007 to April 2008, with the approval of the Institution Review Board at each site.

### Patient selection

Patients selected for the study all provided informed consent. Included in the study were a total of 407 samples from 212 treated or untreated patients in the departments of surgery, hematology, emergency, cardiopulmonary and ICU, who were suspected of having systematic inflammatory response syndrome (SIRS) caused by bacterial or fungal infection, and for whom blood culture was considered to be required for identification of the causative pathogens. Table 2 shows the underlying diseases of the patients studied. The total number of underlying diseases exceeds the total number of enrolled patients since all underlying diseases were counted when a patient had multiple diseases. Of the 407 samples assayed, 277 samples from 156 patients were assessed as SIRS. SIRS was defined as a condition that fulfilled two or more of the following criteria [7]: temperature > 38°C or < 36°C; heart rate > 90 beats per minute; respiratory rate > 20 breaths per minute or PaCO_2 _< 32 mmHg; white blood cell count < 4,000 or > 12,000 cells/μL; or ≥ 10% immature bands.

**Table 2:** Patients' background

		Number of positive samples (%)	
		
	The number of samples	Blood Culture	SeptiFast
Infectious disease	135	22(16.3)	27(20.0)
Blood Stream Infection	104	7(6.7)	5(4.8)
Tumor	51	0(0.0)	4(7.8)
Immune deficiency	33	2(6.1)	5(15.2)
Wound	14	3(21.4)	4(28.6)
Diabetes	48	3(6.3)	7(14.6)
Liver disease	13	0(0.0)	2(15.4)
Kidney disease	11	2(18.1)	2(18.1)
Heart disease	15	1(6.7)	2(13.3)
Pancreatic disease	11	1(9.1)	4(36.3)
Ulcer of the stomach	10	0(0.0)	2(20.0)
Hypertension	9	2(22.2)	2(22.2)
Influenza encephalopathy	7	2(28.6)	1(14.3)
Others	34	4(11.8)	8(23.5)

Total	495	49(9.9)	75(15.2)

### Blood culture analysis

BacT/ALERT 3D (BioMerieux Hazelwood, MO, USA) and BACTEC 9240 systems (Becton, Dickinson Co., Franklin Lakes, NJ, USA) were used for blood culture analysis. Blood administration was followed according to each instruction manual. When the result of blood culture analysis was positive, the sample was identified using each site's identification system. Moreover, we collected the blood culture bottles whose results were positive, and sent them to one commercial laboratory to confirm the validation of the identification microorganisms.

### Blood collection

EDTA-2K vacuum blood collection tubes (Insepack II-D, Sekisui Chemical Co. Ltd., Tokyo, Japan) were used to collect whole blood for Septi*Fast *analysis. Ten milliliters of whole blood were collected for DNA Detection Kit analysis immediately after blood collection for microbial culture. 1.5 mL were used for the assay for DNA Detection Kit. The blood for DNA Detection Kit was stored at -20°C for up to 72 hours before testing. The storage did not affect the assay performance. The detection sensitivity of Septi*Fast *is 30 colony-forming units per mL (CFU/mL), except for coagulase-negative *Staphylococci *(CoNS), *Streptococcus spp*. and *Candida glabrata*, for which the detection sensitivity is 100 CFU/mL [6]. Blood culture was performed at the three sites according to the usual protocol.

### DNA extraction

There are four different Septi*Fast *kits: Septi*Fast *Lys M^Grade^, Septi*Fast *Prep M^Grade^, LightCycler Septi*Fast *M^Grade ^and LightCycler Septi*Fast *mecA M^Grade ^kits (Roche Diagnostics GmbH, Mannheim, Germany). The Septi*Fast*-Lys and Prep kits were used for DNA extraction. The extraction condition for Gram-negative, Gram-positive, and fungi was the same. The assay was performed according to the manufacturer's instructions [6]. To prevent contamination, DNA was extracted in a safety cabinet, M^GRADE ^disposables were used, and DNA extraction and amplification were performed in separate rooms. Negative control extraction was performed concurrently with sample extraction. An internal control (IC) was added to each sample to check for false-negatives.

### Amplification and detection

For detection of Gram-positive and Gram-negative bacteria, and for detection of fungi, 50 μL of each DNA extract was used. The LightCycler Septi*Fast *kit and LightCycler 2.0 (Roche Diagnostics GmbH, Mannheim, Germany) were used for DNA amplification and detection respectively. The amplification region used was an internal transcribed spacer (ITS) region. This region lies between the 16 S and 23 S ribosomal spacer in bacteria and between the 18 S and 5.8 S ribosomal spacer in fungi and is often used to detect bacterial/fungal genes [8,9]. For bacterial/fungal DNA identification after amplification, the DNA of each strain was identified and four different fluorescent nucleotide probes were followed by melting curve analysis. Negative control and the reagent control provided in the kit were used as controls.

### MRSA detection

The presence of MRSA in samples was assayed using the Septi*Fast *mecA kit. MRSA was only assayed in samples in which *S. aureus *was detected, and CoNS was not detected since CoNS-derived mecA genes may compromise MRSA detection [10]. In the samples in which *S. aureus *was detected, but CoNS was not, the presence of mecA genes was confirmed using the LightCycler Septi*Fast *mecA kit and 50 μL of DNA extract, which were prepared using the Septi*Fast *Prep kits.

### Definition of pathogens

It remains difficult to determine whether the organisms detected by the DNA Detection Kit are true pathogens. This also applies, although to a much lesser degree, to conventional blood culture analysis. However, detected organisms were considered to be pathogens if the results of culture tests from samples of the suspected infectious sites coincided with the results of DNA Detection Kit or blood culture analysis. The culture data of sputum, urine, pus and drainage fluid were used to define the pathogens.

**Figure 1:**
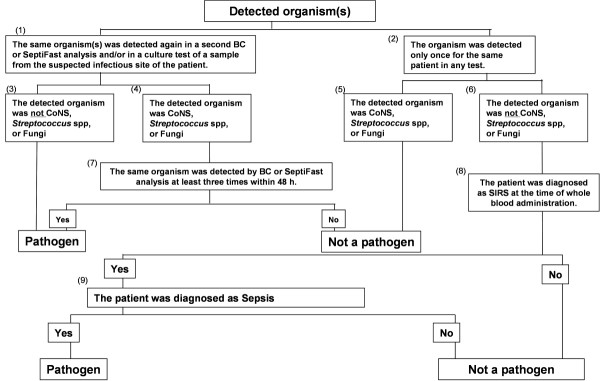
**Flowchart for pathogen decision**.

A decision as to whether an identified organism was a pathogen was taken based on the decision tree shown in Figure 1. Thus, when the same organism was detected by both DNA Detection Kit and blood culture analysis, the detected organism was considered an infectious pathogen. If there was a discrepancy between the organism that was detected by Septi*Fast *analysis and that detected by blood culture analysis, or if an organism was only detected in one of these tests, then other samples taken from the infection site were analyzed. If this second culture test of the suspected infectious site revealed the presence of the same organism, this organism was considered to be a pathogen. If the microbial strain was only detected once for a sample, we then checked the second culture results in the suspected infectious sites. If this result identified the same strain as that identified by Septi*Fast *analysis then it was decided that this strain was a pathogen. However, if the strain was still only detected in some of the assays, we next determined if the patient involved suffered from sepsis. Sepsis is defined as SIRS caused by infection. The definition of sepsis that we used was based on the International Sepsis Forum Definition of Infection at the ICU Consensus Conference [7]. However, if the underlying disease is acute lymphoma leukemia (ALL), malignant lymphoma (ML), or acute myelogenous leukemia (AML), the definition of infection is defined as the ability to detect infectious organisms by blood culture analysis. If the patient was not defined as having sepsis when whole blood was administered to the patient, we decided that the strain detected by subsequent DNA Detection Kit or blood culture analysis was not a pathogen.

Samples were defined as negative for pathogens if a pathogen could not be detected by any method of analysis within seven days, and if another type of culture test did not detect this pathogen but could detect other organisms.

CoNS bacteria, which are represented by the *Staphylococcus epidermidis (S. epidermidis) *and *Streptococcus spp*. are indigenous bacteria and often cause contamination in assays of pathogens. Therefore, when CoNS or *Streptococcus spp*. were detected by blood culture and Septi*Fast *analysis, the following criteria were applied to define whether these strains represented a pathogenic infection**: **(1) Tests were performed at least twice within 48 hours before and after CoNS were detected by blood culture or Septi*Fast *analysis; (2) CoNS or *Streptococcus spp*. were detected in two different blood culture tests that were separately performed twice within 48 hours; and, (3) CoNS or *Streptococcus spp*. were detected twice or more in tests that were performed three times [11-15]. If a sample's results met any of these three criteria, then the sample was evaluated as a pathogen.

The distinction between pathogen and contamination was also determined for CoNS or *Streptococcus spp*. from the crossing point (Cp) obtained using the LightCycler analysis software v4.05. The Cp represents the point in the amplification cycle where the amplification curve crosses the detection threshold. When CoNS or *Streptococcus spp*. were detected using the LightCycler analysis software v4.05, a Cp of less than 20 was defined as indicating a pathogen and a Cp of over 20 was defined as contamination by checking the amplification curve.

### Antibiotic administration survey

Antibiotic administration to patients at the time of blood collection was checked and it was confirmed that the spectrum of the antibiotic used corresponded to the organism detected in the blood analyses. The antibiotic spectra were determined based on information regarding susceptible organisms provided by the pharmaceutical company that marketed each antibiotic.

### Statistical analysis

McNemar's test was conducted at a significance level of 5% to compare DNA Detection Kit and blood culture detection of pathogens. A two-sample test for equality of proportions was conducted at a significance level of 5% to compare detection of pathogens when DNA Detection Kit and blood culture results were combined.

## Results

### Correlation between SeptiFast and blood culture analyses

The patients consisted of 137 males and 75 females. Table 2 demonstrates that Septi*Fast *analysis detected more organisms in patients than blood culture analysis.

**Figure 2:**
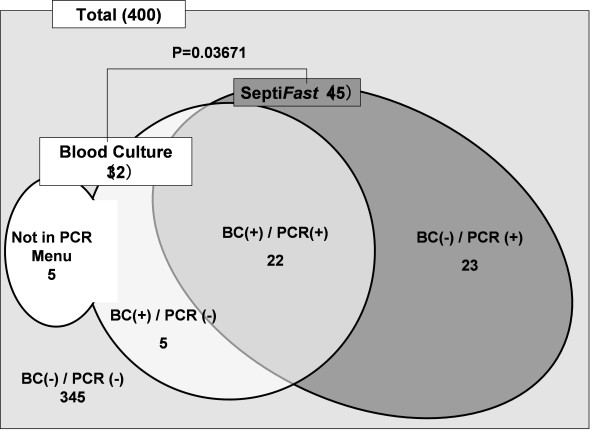
**Summary of the number of pathogens detected by Septi*Fast *(PCR) and/or blood culture analysis**.

Figure 2 shows the correlation between blood culture and Septi*Fast *analyses. No specific pathogen could be identified in seven of the samples (by either method). These samples were therefore eliminated from the study since they did not meet the definition of sepsis, leaving a total of 400 samples that were evaluated. The DNA Detection Kit identified a pathogen in 11.3% (45/400) of the samples, and blood culture analysis identified a pathogen in 8.0% (32/400) of the samples. The difference between positive and negative results for each assay was statistically different, as measured using McNemar's test (*P *< 0.04). Of the 22 samples in which pathogens were detected by both blood culture and DNA Detection Kit analyses, there was one sample in which there was a discrepancy in the pathogen that was detected. In this sample, *E. faecium *was detected by blood culture analysis but *E. coli *was detected by Septi*Fast *analysis. We confirmed *E. coli *and *E. faecium *were detected from the other sample of the same patient. Thus, it was decided that both organisms were pathogens. Table 3 summarizes the number of samples in which each of the listed organisms was identified. The detected pathogen is total 56 because we count both *E. coli *and *E. faecium *as pathogens.

**Table 3:** Pathogens detected by Septi*Fast *and blood culture analyses

	Strain detected		
	
Pathogen	Only by BC	Only by Septi*Fast*	Both methods
*S.aureus *(MSSA)	0	3	3
*S.aureus *(MRSA)	2	0	4
*S.pneumoniae*	0	1	0
Streptococcus spp.	0	2	1
*Enterococcus faecalis*	0	1	0
*Enterococcus faecium*	2	0	0
*Enterobacter aerogenes/cloacae*	0	3	1
*Escherichia coli*	0	3	9
*Klebsiella pneumoniae/oxytoca*	1	5	1
*Pseudomonas aeruginosa*	1	4	1
*Candida albicans*	0	1	0
*Candida tropicalis*	0	1	1
Sub-total	6	24	21
Not detectable by SeptiFast	5	0	0

Total	11	24	21

Twenty-three pathogens were detected by Septi*Fast *only. All of the pathogens detected only by DNA Detection Kit were identified as the same organism from the other culture. Pathogens were detected in 10 of the samples only by blood culture analysis. The organisms identified in five of these samples, *Bacteroides spp*., Gram-positive rod and *Morganella morganii *(in 2, 2 and 1 samples respectively), are not listed as organisms that can be detected by Septi*Fast *analysis. Of the remaining five samples, MRSA was detected in two of the samples and *Pseudomonas aeruginosa, Klebsiella and Enterococcus faecium *were each detected in one of the remaining samples.

**Figure 3:**
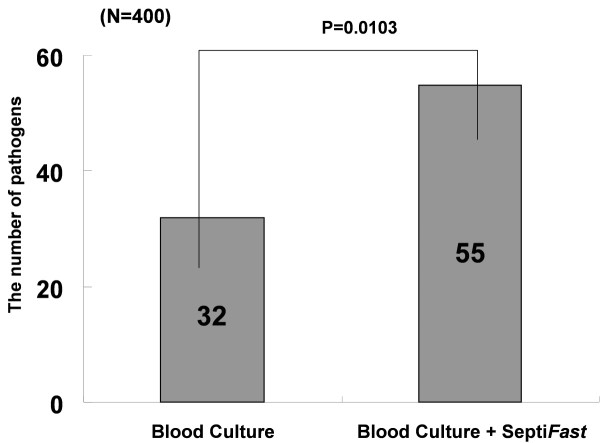
**Comparison of pathogen detection by blood culture analysis and by blood culture combined with Septi*Fast *analysis**.

Figure 3 shows the change in the number of samples testing positive for a pathogen when the positive results of blood culture and Septi*Fast *were combined. This figure demonstrates that the number of samples testing positive in SIRS samples only, increased from 9.0% (35/387) to 16.0% (62/387) when organisms that were detected by blood culture analysis, and those that were detected by Septi*Fast *analysis, were combined. A significant difference in the number of positive samples from the combined tests compared to that in the individual tests was observed using a two-sample test for equality of proportions (*P *= 0.01).

### MRSA detection

In this study, 12 samples tested positive for *S. aureus *as a pathogen. Of these 12 samples, 10 were detected by Septi*Fast *analysis and 9 were detected by blood culture analysis. However, while blood culture analysis detected MRSA in six samples, Septi*Fast *analysis only detected MRSA in four samples. Two samples were diagnosed as being infected by MRSA based on the analysis shown in the decision tree (Figure 1).

### The affect of antibiotics administration

As shown in Figure 2, a total of 55 pathogens were detected by Septi*Fast *or blood culture analysis. Of these 55 samples, 40 samples (72.7%) were from patients which had been administered antibiotics and 32 of these 40 samples (80.0%) were from patients that had been administered antibiotics that matched the spectra of the antibiotics. These 32 samples were evaluated for the presence of pathogens by blood culture and DNA Detection Kit. Septi*Fast *analysis detected pathogens in 21 samples, while blood culture analysis detected pathogens in 10 samples, indicating that DNA Detection Kit analysis detected significantly more pathogens than blood culture analysis (*P *= 0.02) under these conditions. These data further suggest that detection of pathogens by blood culture analysis was affected by antibiotics, since there were 15 samples in which pathogens were detected only by DNA Detection Kit, but not by blood culture analysis. Of the four samples in which pathogens were detected by blood culture analysis but not by Septi*Fast *analysis, one of these samples was identified as containing the pathogen *Bacteroides caccae*, which is an organism that cannot be detected by Septi*Fast*.

## Discussion

Sepsis is an infection frequently found in transplant patients, in patients with hematological neoplasms or in patients admitted to an intensive care unit (ICU) following surgery. Rapid pathogen identification and the appropriate chemotherapy are important to improve patient prognoses. Definitive identification of bacterial species with a microarray platform was highly expected [16]. A rapid pathogen detection and diagnosis kit for sepsis called Septi*Fast *has recently been developed [17]. This kit will reduce the turn-around time to detect pathogens. Louie *et al*. surveyed Septi*Fast *pathogen detection times using samples from seven patients and reported that the average pathogen detection time was 6.54 ± 0.27 hours [18].

As shown in Figure 2, we confirmed that Septi*Fast *analysis significantly detected more pathogens than blood culture analysis. However, a discrepancy between the results of Septi*Fast *and blood culture analysis was noted for one sample. In this sample, *E. coli *was detected by Septi*Fast *analysis, but *E. faecium *was detected by blood culture analysis. We rechecked the presence of these organisms in more samples from the patient and found that *E. coli *had been detected by Septi*Fast *and blood culture analysis in samples that were submitted three days before and that *E. faecium *was detected by blood culture analysis two days after. Therefore, it was considered that bacterial translocation had occurred in this patient. In 23 of the samples assayed in this study, pathogens were only identified by DNA Detection Kit. One possible reason why a pathogen was not detected in these samples by blood culture analysis was that blood culture analysis might have been affected by the treatment of the patients with antibiotics. Indeed, 15 of these 23 patients (65.2%) had been administered antibiotics appropriate for the pathogen in question. In 10 samples in this study, pathogens were detected only by blood culture analysis. The reason that Septi*Fast *analysis could not detect these pathogens was considered to be that the concentration of these pathogens was very low and therefore it was outside the limit of detection (LOD) of Septi*Fast *analysis.

Of the 12 samples that tested positive for *S. aureus *in this study, 10 were detected by DNA Detection Kit but only 9 were detected by blood culture analysis. However, as shown in Table 3, blood culture analysis detected MRSA in six samples whereas Septi*Fast *detected MRSA in only four samples. This discrepancy may be caused by the LOD gap mentioned above. Thus, the sensitivity of detection of *S. aureus *and the mecA gene was 30 CFU/mL for the Septi*Fast *assay system, but the LOD is 7.7 CFU/mL for *S. aureus *and 24.2 CFU/mL for mecA genes [19]. Therefore, the reason why MRSA could not be detected by Septi*Fast *analysis, but could be detected by blood culture analysis, may be due to a difference in the detection sensitivity of these two assay systems.

As shown in Table 4, Septi*Fast *analysis detected more pathogens than blood culture analysis when antibiotics had been administered to the patients. Although the antibiotics used prevented the growth of organisms in blood culture analysis, it appeared that DNA Detection Kit could detect pathogens with relatively little interference by antibiotics. Our results are in agreement with the information provided by the Septi*Fast *manufacturer that antibiotics do not interfere with Septi*Fast *detection of pathogens [6]. These data suggest that Septi*Fast *will have clinical utility for analysis of pathogens in patients with a background of unknown pre-treatment of antibiotics due to being referred from other hospitals, and for patients receiving antibiotics before blood collection for testing due to the severity of their condition. Another clinical benefit of Septi*Fast *is that the test result is achieved faster than the result of blood culture analysis, and thus will allow a speedier de-escalation from a broad- to a narrow-spectrum antibiotic. According to the "Surviving Sepsis Campaign Guidelines (SSCG) 2008", antibiotic administration within an hour is recommended in patients suspected of having severe sepsis [20]. Therefore, the use of the DNA Detection Kit, whose pathogen detection ability is not susceptible to the effects of antibiotic administration, should contribute to implementation of these guidelines.

**Table 4:** Comparison of pathogen detection by Septi*Fast *and blood culture analyses following treatment with the antibiotic appropriate to the pathogen

		Blood Culture		
			
		Positive	Negative	Total
SeptiFast	Positive	6	15	21
				
	Negative	4^a^	7	11

Total		10	22	32

^a ^One of these four pathogens was a pathogen that is not detectable by Septi*Fast*.

In Japan, blood culture analysis is the gold standard of pathogen analysis when sepsis is suspected. However, it is anticipated that if Septi*Fast *analysis is introduced, it will facilitate the selection of antibiotics based on EBM due to earlier pathogen detection and to the detection of more pathogens. DNA Detection Kit analysis cannot replace blood culture analysis because it cannot detect all sepsis pathogens. However, by combining Septi*Fast *and blood culture analyses, the detection rate of pathogens will significantly increase. A faster detection rate will be especially useful for SIRS patients since more precise sepsis treatment will become feasible. Since the use of the DNA Detection Kit requires skilled clinical laboratory technicians and suitable facilities, the kit should be used in university hospitals where severe sepsis patients are gathered.

The extended duration of surgical antibiotic prophylaxis for up to seven days and multicoverage for empiric therapy of suspected sepsis is performed in Japan. Thus, our results are not easily applicable to other regions since the diagnostic value of conventional blood culture systems in this study may have been decreased by very frequent previous antibiotic exposure.

## Conclusions

Although DNA Detection Kit analysis could not detect all sepsis pathogens, Septi*Fast *analysis did detect potentially important pathogenic DNA that could not be detected by blood culture analysis. Simultaneous testing of samples from patients with demonstrated SIRS using blood culture analysis and DNA Detection Kit showed a high pathogen detection rate. This rapid multiplex pathogen detection system complemented traditional culture-based methods and offered some added diagnostic value for the timely detection of causative pathogens, particularly in antibiotic pre-treated patients. Furthermore, the ability of Septi*Fast *analysis to identify pathogens when the background of antibiotic administration is unknown may allow a change to narrower-spectrum antibiotics. The combined data suggest that Septi*Fast *may ultimately contribute both to the improvement of patient safety and to future medical economic efficiency. Clearly, adequately designed intervention studies are urgently needed to prove its clinical effectiveness in improving appropriate antibiotic selection and patient outcomes.

## Key messages

• This rapid multiplex pathogen detection system showed a higher pathogen detection rate in comparison with blood culture analysis.

• This system offered some added diagnostic value for the timely detection of causative pathogens, particularly in antibiotic pre-treated patients.

• However, the well designed intervention studies are urgently needed to prove the clinical effectiveness.

## Abbreviations

ALL: acute lymphoma leukemia; AML: acute myelogenous leukemia; Cp: crossing point; EBM: evidence-based medicine; IC: internal control; ICU: intensive care unit; ITS: internal transcribed spacer; LOD: limit of detection; ML: malignant lymphoma; MRSA: methicillin-resistant *Staphylococcus aureus*; PCR: polymerase chain reaction; SIRS: systemic inflammatory response syndrome; SSCG: Surviving Sepsis Campaign Guidelines.

## Competing interests

The authors declare that they have no competing interests.

## Authors' contributions

KY, YK, SK, KS, SA, HG and MK carried out the molecular genetic studies, participated in the sequence alignment and drafted the manuscript. MT, KT, SK, MS, HS and TS participated in the sequence alignment. OT, AM, YI, SO, NA and SH participated in the design of the study and performed the statistical analysis. HO, AI, NH, JT, MM, YK and YS conceived of the study, and participated in its design and coordination and helped to draft the manuscript. All authors read and approved the final manuscript.
